# Inhibition of ULK1 promotes the death of leukemia cell in an autophagy irrelevant manner and exerts the antileukemia effect

**DOI:** 10.1002/ctm2.282

**Published:** 2021-01-12

**Authors:** Wei Yang, Yunlei Li, Shuai Liu, Weimin Sun, Hualan Huang, Qiqing Zhang, Jie Yan

**Affiliations:** ^1^ The Second Affiliated Hospital The State Key Laboratory of Respiratory Disease Guangdong Provincial Key Laboratory of Allergy & Clinical Immunology Guangzhou Medical University Guangzhou China; ^2^ Institute of Biomedical Engineering The Second Clinical Medical College (Shenzhen People's Hospital) of Jinan University Shenzhen China; ^3^ Department of Laboratory Shenzhen Sami International Medical Center (Shenzhen Fourth People's Hospital) Shenzhen China; ^4^ The Second Affiliated Hospital Guangzhou Medical University Guangzhou China

Dear Editor,

Because of disease relapse and drug resistance, patients with various subtypes of acute myeloid leukemia (AML) suffer low rate of 5‐year survival. Although our understanding of the molecular pathogenesis of AML has made progress, there is a real need to develop new therapeutic targets and drugs to target leukemia for improving the treatment of patients with AML.^1^

As the homologous of autophagy‐related 1 (ATG1), the classic function of unc‐51‐like kinase 1 (ULK1) is to initiate autophagy in response to nutritional starvation.^2^, ^3^ Recently, increasing evidence suggests that abnormal expression of ULK1 is associated with the progression of various solid tumors and poor prognosis. Several small molecules targeting ULK1 have been developed to inhibit the growth of related tumors, such as SBI‐0206965 for clear cell renal cell carcinoma^4^ and non‐small cell lung cancer^5^ intervention. All these indicate that ULK1 could be a potential target for certain tumor treatments, but its efficacy in AML still remains unknown.

Here, we analyzed the mRNA expression level of *ULK1* in AML samples from The Cancer Genome Atlas (TCGA) available database. The results showed that the level of *ULK1* in AML increased to some extent (Figure 1A), which was associated with poor prognosis of AML (Figure 1B). The *ULK2*, *ULK1* homologous gene, was also increased in AML samples (Figure 1C). However, the high expression of ULK1 and weak expression of *ULK2* were detected in leukemia cell lines (Figure 1E and Table S1). In addition, the expression of ULK1 in primary AML samples is significantly increased compared with healthy control, whereas failed to detect the expression of ULK2 (Figure 1F). These data suggested that ULK1 but not ULK2 may be an important factor involved in the process of AML.

**FIGURE 1:**
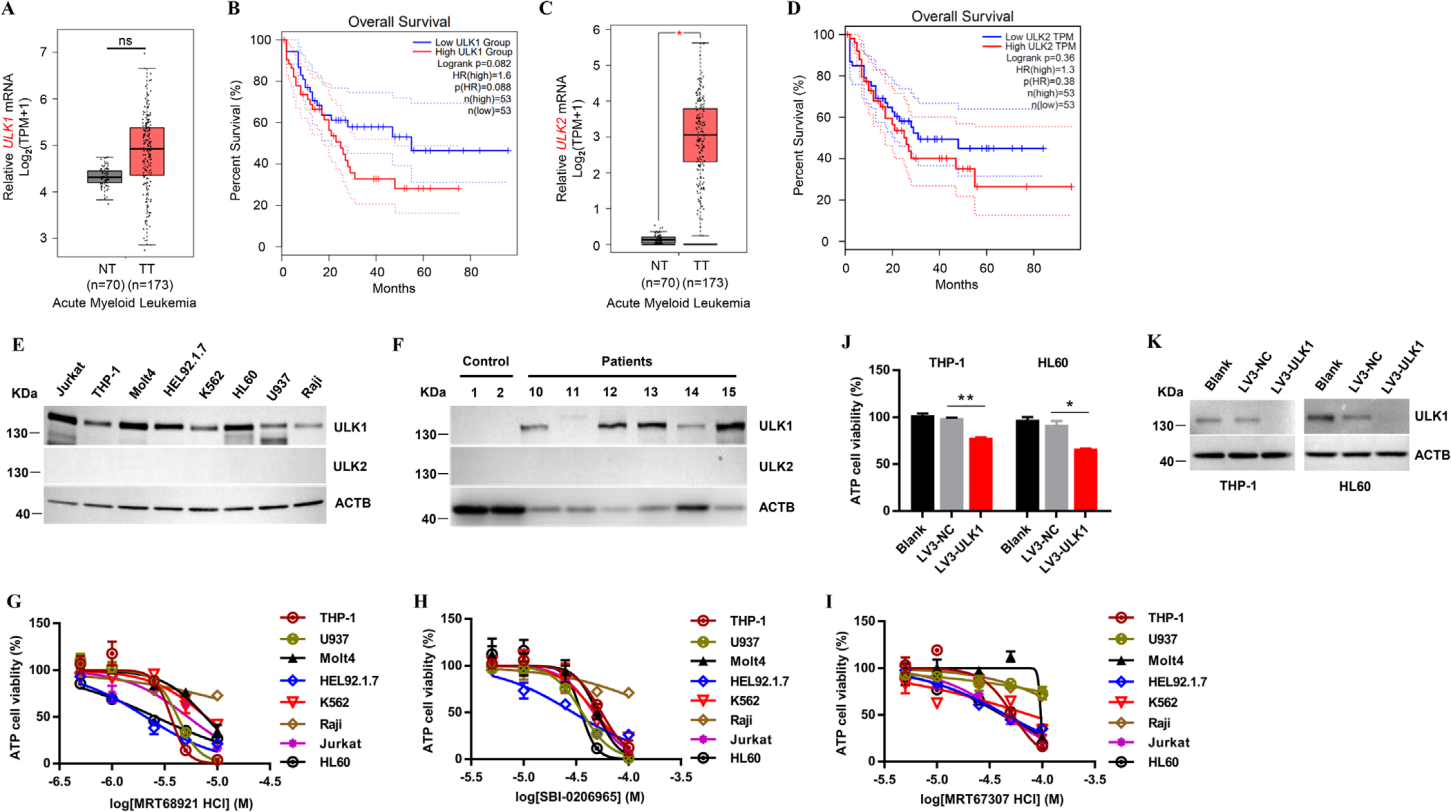
Targeted ULK1 inhibition induced leukemia cells death. A, The relative mRNA expression of *ULK1* in acute myeloid leukemia (AML) patients was upregulated when compared with that in healthy control, based on analysis of The Cancer Genome Atlas (TCGA) data. NT, normal tissue. TT, tumor tissue. B, Kaplan‐Meier analysis of overall survival for AML patients relative to expression levels of *ULK1*. Patients were stratified as low and high expression of *ULK1* mRNA. *GAPDH* was used as housekeeping gene for normalization. *P* value = .082. C, The relative mRNA expression of *ULK2* in acute myeloid leukemia (AML) patients was upregulated when compared with that in healthy control, based on analysis of The Cancer Genome Atlas (TCGA) data. D, Kaplan‐Meier analysis of overall survival for AML patients relative to expression levels of *ULK2*. Patients were stratified as low and high expression of *ULK2* mRNA. *GAPDH* was used as housekeeping gene for normalization. *P* value = .36. Leukemia cells were treated as indicated doses and inhibitors for 20 h, then ATP‐based cell viability was analyzed by Enhanced ATP Assay kit (Beyotime). E, ULK1 and ULK2 protein expression was analyzed in leukemia cell lines by western blot. Forty micrograms protein samples derived from leukemia cells were loaded for western blot analysis. ACTB (β‐actin) as internal control. F, Six cases of primary AML cells were purified directly from peripheral blood of patients diagnosed with leukemia, and two cases of PBMC were derived from healthy donator. ULK1, ULK2, and ACTB as controls were analyzed by immunoblotting. Twelve micrograms primary AML cell protein samples and 20 µg normal PMBC protein samples were loaded for western blot analysis. G‐I, Leukemia cells death induced by three ULK1‐specific inhibitors MRT68921 HCl (G), SBI‐0206965 (H), and MRT67307 HCl (I). J, Knockdown of ULK1 in THP‐1 and HL60 cells with lentiviral for 72 h, and then ATP‐based viability was analyzed by Enhanced ATP Assay kit. K, ULK1 knockdown efficiency in THP‐1 and HL60 cells was determined by western blotting. Data in (J, G, H, I) were presented as mean ± SEM. All of the results represented at least three independent experiments. *P<.05, **P<.01 between the indicated groups

To further analyze the potential role of ULK1, three specific commercial inhibitors of ULK1 were used to treat leukemia cell lines. Compared with MRT67307 HCl and SBI‐0206965, MRT68921 HCl exerts a stronger killing effect (Figure 1G‐I and Table S2). It is consistent with the phenomenon of *ULK1* knockdown with lentivirus (Figure 1J, K). These results indicate that ULK1 plays an important role in leukemia cell survival. We further tested the killing effect of MRT68921 HCl on primary leukemia tumor cells derived from AML patients (see Table S3 for specific classification). Results showed that MRT68921 HCl induced obvious death of primary tumor cells through Annexin V/PI staining and FACS analysis (Figure S1). Taken together, targeting ULK1 with MRT68921 HCl could efficiently induce the death of leukemia cell lines and primary leukemia cells.

We further tested the *in vivo* antitumor effect of MRT68921 HCl in THP‐1 leukemia model mice. THP‐1 cells stably transfected with luciferase were used to construct a mouse leukemia model,^6^ and treated with MRT68921 HCl or vehicle for 3 weeks. The tumor burden in the mice was evaluated by the bioluminescence, and the results showed that with the treatment of MRT68921 HCl, the tumor burden in the treated group decreased by 36.5%, 84.8%, and 69.2% at 2, 3, and 4 weeks, respectively (Figure 2A, B), and the average survival time extended for 6.5 days (Figure 2C). In addition, less infiltrated leukemia cells in the spleen, liver, and bone marrow were dected on day 20 after MRT68921 HCl‐treatment (Figure 2D). FACS analysis of cell suspension revealed that the proportion of human CD45 positive tumor cells in the liver and spleen significantly reduced on day 21, which was consistent with immunohistochemical staining in tissue slides (Figure 2E). All of these results showed that MRT68921 HCl exerts antitumor activity in mice.

**FIGURE 2:**
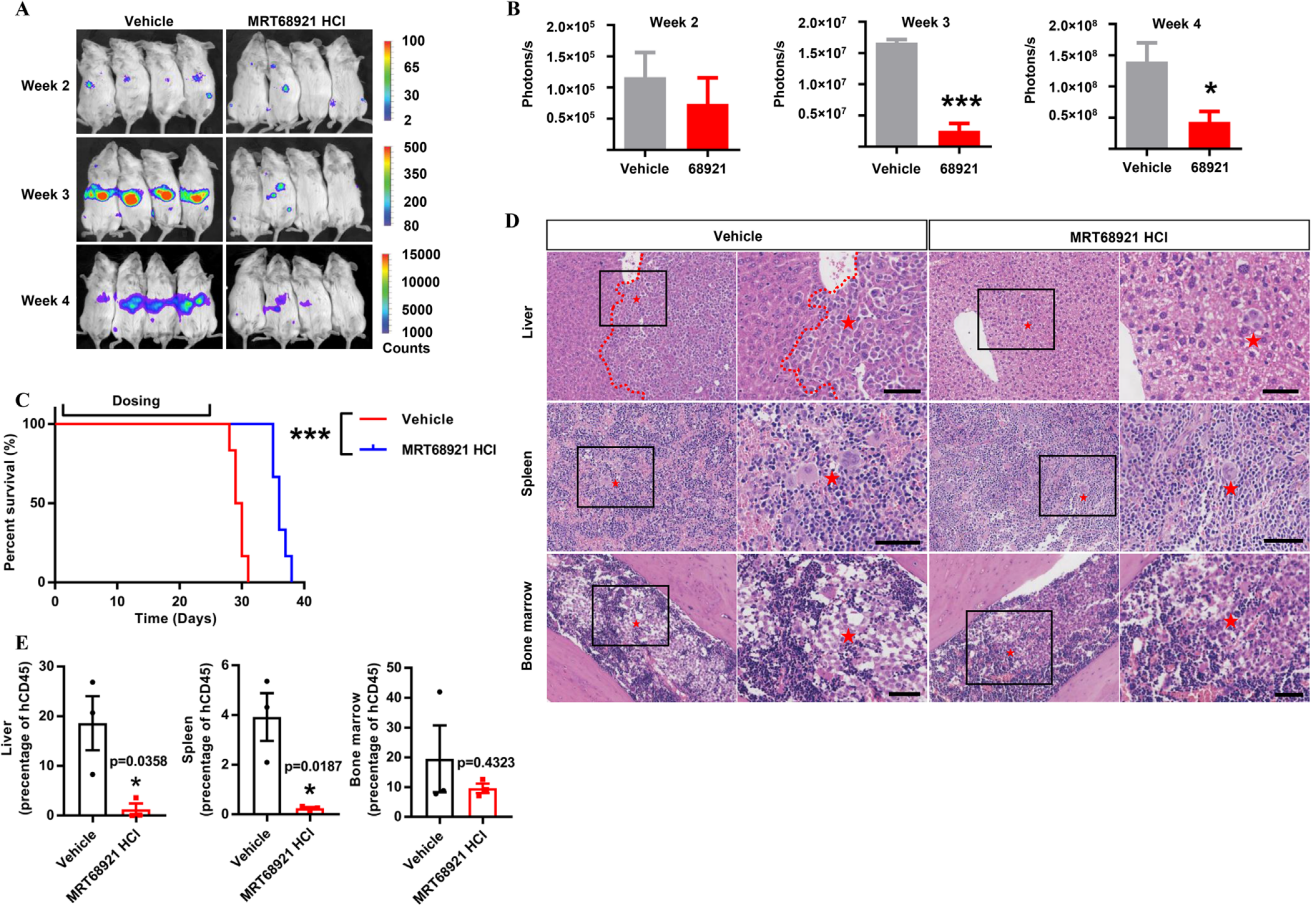
MRT68921 HCl inhibits AML progression *in vivo*. THP‐1 cells stably expressing the luciferase gene were injected into mice via the tail vein. A, Bioluminescence imaging of MRT68921 HCl (30 mg/kg) and vehicle‐treated mice over the time course of study (n = 4 mice per group). B, Bioluminescence quantification of MRT68921 HCl‐ and vehicle‐treated mice in A. C, Kaplan‐Meier survival analysis (n = 6 mice per group, *P* = .0005, two‐sided log‐rank test). D, Photomicrographs (n = 3) of hematoxylin and eosin‐stained sections of mouse live, spleen, and bone marrow from the two groups on treatment day 20. E, FACS analysis of the percentage of human CD45^+^ cells in liver, spleen, and bone marrow from mice after 21 days treatment of vehicle and MRT68921 HCl. Data shown as mean ± SEM. *P<.05, ***P<.001 between the indicated groups. Scale bar, 50 µM

We further analyzed the molecular mechanism of the cell death after ULK1 inhibition. First, the death morphology showed that THP‐1 swelled but HL‐60 appeared to be apoptotic bodies (Figure 3A), which suggested different death manner in these two cell types. Western blot results showed that MRT68921 HCl induced GSDME cleavage in THP‐1 cells, but PARP cleavage in HL‐60 cells (Figure 3B). Furthermore, MRT68921 HCl significantly induced the activity of caspase‐3 and caspase‐8 in both THP‐1 and HL60 cells (Figure 3C). All of these results proved that THP‐1 cells suffered from pyroptosis by caspase‐3‐GSDME activation, but HL‐60 cells experienced caspase‐3‐PARP activated apoptosis due to the absence of GSDME expression.

**FIGURE 3:**
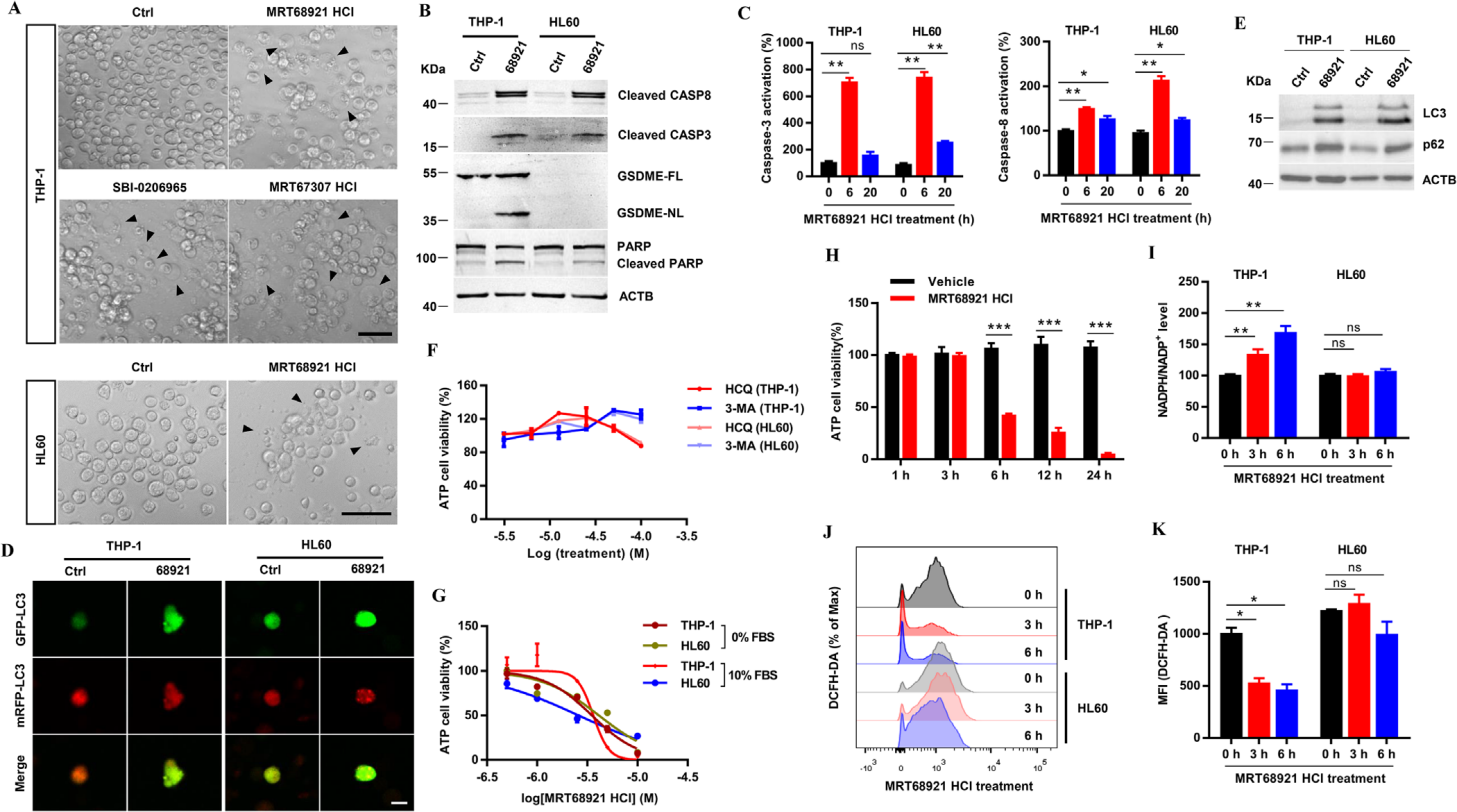
MRT68921 HCl induced leukemia cells death by activating caspase‐3/8 pathway in an autophagy and reactive oxygen species independent manner. A, Static bright field images of THP‐1 and HL60 cells treated with indicated ULK1 inhibitors compared to control group for 20 h. Arrows indicate dying cells. B, Cleaved caspase‐8, cleaved caspase‐3, GSDME‐FL, GSDME‐NL, PARP, cleaved PARP, and ACTB as controls were analyzed by immunoblotting. C, Caspase‐3 and caspase‐8 activation in THP‐1 and HL60 cells treated with MRT68921 HCl (5 µM) for 6 and 20 h were determined by Caspase‐3 and Caspase‐8 Activity Assay Kit (Beyotime), respectively. Scale bars, 50 µm. D, Confocal images of cells expressing mRFP‐GFP‐LC3 that were treated without or with MRT68921 HCl. Scale bar, 10 µm. E, LC3 and p62 in THP‐1 and HL60 treated with MRT68921 HCl (5 µM) were analyzed by immunoblotting. ACTB as an internal control. F, Over dose of autophagy inhibitors 3‐MA and HCQ had no obvious effect on THP‐1 and HL60 cell death. G, THP‐1 and HL60 cells treated with different dose of MRT68921 HCl for 20 h under 0% and 10% FBS culture conditions, and then ATP‐based cell viability was analyzed by Enhanced ATP Assay kit (Beyotime). H, Time course of cell death of THP‐1 cells treated with MRT68921 HCl. I and J, THP‐1 and HL60 cells treated with MRT68921 HCl for 3 and 6 h, and then NADPH/NADP^+^ ratio was determined using NADP^+^/NADPH Assay Kit with WST‐8 (I), and ROS level was determined by fluorescent DCFH‐DA assay and FACS analysis (J). K, Quantitative analysis of DCFH‐DA median fluorescence intensity (MFI) for ROS level as described for panel J. Data in (C, F, G, H, I and K) were as mean ± SEM. All of the results repreasented at least three independent experiments. *P<.05, **P<.01, ***P<.001 between the indicated groups

As a key role in autophagy, ULK1 blocking and autophagy interference is one of the important strategies for solid tumor treatment. However, the results showed that GFP‐LC3 and mRFP‐LC3 puncta were increased by MRT68921 HCl (Figure 3D), and the level of LC3‐II increased and p62 accumulated (Figure 3E) in THP‐1 and HL60 cells, indicating that MRT68921 HCl induced autophagy in parallel with inhibiting autophagic flux in AML cells. In addition, neither 3‐MA nor HCQ has obvious effect on the cell viability at concentrations above IC_50_ (Figure 3F), and no difference in the IC_50_ values of MRT68921 HCl was detected under serum starvation and normal nutrition condition (Figure 3G). These results suggested that ULK1 inhibition‐induced cell death in leukemia is not associated with autophagy inhibition.

It was reported that PPP activity depends on ULK1 kinase.^7^ ULK1 kinase depletion could increase ROS level and decrease the ratio of NADPH/NADP^+^ that finally promote caspase‐3‐mediated apoptosis of carcinoma.^4^ To verify the ROS‐dependent mechanism of ULK1 inhibitors induced cell death, the NADPH/NADP^+^ ratio and ROS levels were detected after MRT68921 HCl treatment. In contrast, MRT68921 HCl decreased ROS level and increased NADPH/NADP^+^ ratio selectively in THP‐1, but not in HL60 (Figure 3H‐K). Taken together, as the specific inhibitor of ULK1, MRT68921 HCl‐induced leukemia cell death is independent of reactive oxygen species. Further experiments are needed for the specific mechanism of MRT68921 HCl‐induced leukemia cell death.

In summary, our results show that the ULK1 inhibitor MRT68921 HCl has a significant antitumor effect *in vitro* and *in vivo*, in an autophagy and reactive oxygen species in independent manner, indicating it as a potential application in the AML treatment.

## CONFLICT OF INTEREST

The authors declare no conflict of interest.

## AUTHOR CONTRIBUTIONS

Jie Yan is the principal investigator responsible and take full responsibility for the paper. Wei Yang contributed to generation of hypothesis and experimental design. Wei Yang, Yunlei Li, Shuai Liu, Weimin Sun, and Hualan Huang contributed to the execution of experiments. Wei Yang, Yunlei Li, and Shuai Liu contributed to data analyses and literature review. Wei Yang, Qiqing Zhang, and Jie Yan were responsible for writing the manuscript with input from all authors. Qiqing Zhang and Jie Yan revised the manuscript.

## Supporting information

Supporting Information

Supporting Information
