# Supplementary material for: Inhibition of ULK1 promotes the death of leukemia cell in an autophagy irrelevant manner and exerts the antileukemia effect
Source: Clin Transl Med. 2021 Jan 12;11(1):e282. doi: 10.1002/ctm2.282 (PMC7803353; doi:10.1002/ctm2.282)
Supplement: Supplementary file 1 — Supporting Information [file CTM2-11-e282-s001.docx]

**Supplementary Figure 1** **MRT68921 HCl induced the death of primary leukemia cells derived from leukemia patients.** Primary leukemia cells were purified directly from peripheral blood of patients diagnosed with leukemia. Cells were treated with MRT68921 HCl (5 μM) or vehicle (0.9% NaCl) for 24 h. **A**, Cell viability were determined by Annexin V/PI staining and analyzed by fluorescence-activated cell sorting (FACS) analysis. **B**, The proportion of Annexin V-stained cells in MRT68921 HCl-induced groups compared to vehicle-treated groups. Data represents means ± SEM, from two or three technical replicates.

**Supplementary Table 1** *ULK1* and *ULK2* mRNA expression was detected in leukemia cell lines by Q-PCR. β-actin was used as endogenous control transcripts for normalization of the target transcripts. Relative gene expression was analyzed using the 2-∆∆Ct method.

| **Cell line** | **ΔCt (*ULK1*)**  **（normalized to ACTB）** | **ΔCt (*ULK2*)**  **（normalized to ACTB）** |
| --- | --- | --- |
| Jurkat | 9.09 | 22.25 |
| THP-1 | 12.03 | 15.17 |
| Molt4 | 7.25 | 20.83 |
| HEL92.1.7 | 8.6 | 20.05 |
| K562 | 7.4 | 18.91 |
| HL60 | 8.39 | 15.09 |
| U937 | 10.41 | 22.19 |
| Raji | 9.66 | 19.22 |

**Supplementary Table 2** IC_50_ value of ULK1 inhibitor-induced leukemia cells death, calculated by GrapdPad software.

| **IC_50_（μM）** | **MRT68921 HCl** | **SBI-0206965** | **MRT67307 HCl** |
| --- | --- | --- | --- |
| THP-1 | 3.6 | 55.3 | 52.5 |
| U937 | 4.2 | 36.1 | 809.8 |
| Molt4 | 7.7 | 50.2 | ～96.8 |
| HEL92.1.7 | 2.1 | 29.5 | 45.4 |
| K562 | 7.8 | 47.9 | 76.3 |
| Raji | 36.6 | 236.1 | 207.9 |
| Jurkat | 5.2 | 46.4 | 43.3 |
| HL60 | 2.6 | 35.4 | 41.7 |

**Supplementary Table 3** Primary leukemia cell samples used in this study. All samples were derived from patients who had been diagnosed leukemia in the laboratory of the Second Affiliated Hospital of Guangzhou Medical University. N/A, not available.

| **Sample ID** | **Malignancy** | **Sex** | **Age** | **WBC 10^9^/L** | **Blasts (%)** | **Cytogenetics** | **Mutations** |
| --- | --- | --- | --- | --- | --- | --- | --- |
| Patient-1 | B-ALL | M | 59 | 1.36 | 69.5 | N/A | N/A |
| Patient-2 | T/B lymphoma | M | 39 | 11.95 | 94.5 | N/A | N/A |
| Patient-3 | CML | F | 73 | 213.05 | 0.8 | 46,XX,t(9,22)(q34,q11.2) | N/A |
| Patient-4 | M4 AML | M | 65 | 6.45 | 12 | 46,XY,t(8,21)(q22,q22) | AML/ETO |
| Patient-5 | MDS-RAEB | F | 57 | 5.08 | 82 | N/A | DNMT3A,TET2,ASXL1,IDH1,IDH2,E2H2 |
| Patient-6 | M3 AML | F | 22 | 1.26 | 72 | N/A | PML/RARɑ |
| Patient-7 | M5 AML | M | 66 | 106.02 | N/A | N/A | FLT3,IDH2,NPM1 |
| Patient-8 | B-ALL | F | 56 | 14.33 | N/A | N/A | N/A |
| Patient-9 | M4 AML | F | 37 | 4.1 | 44.7 | N/A | N/A |
| Patient-10 | M2a | M | 37 | 105.4 | N/A | N/A | N/A |
| Patient-11 | M1 | M | 53 | 172.3 | 65 | N/A | IDH1,NRAS,BCOR |
| Patient-12 | M4 | M | 30 | 36.06 | 33.5 | N/A | CEBPA,WT1,IKZF1 |
| Patient-13 | M3 | F | 23 | 28.4 | 2.5 | N/A | IDH2,NPM1,FLT3 |
| Patient-14 | M2a | F | 45 | 220.9 | 59.6 | N/A | FLT3,DNMT3,ETV6 |
| Patient-15 | M5 | M | 67 | 2.22 | 48.5 | N/A | FLT3,SRSF2,TET2,STAG2 |

**Materials and Methods**

**Reagents and antibodies**

ULK1 inhibitors, MRT68921 HCl，MRT67307 HCl，and SBI-0206965 were obtained from TargetMol (Wellesley, MA). Z-VAD (OMe)-FMK (HY-16658) was purchased from MedChemExpress (Monmouth, NJ). Primary antibodies used were as follows: caspase-3 (A0214, ABclonal Biotechnology, Wuhan, China), cleaved caspase-3 (#9664, Cell Signaling Technology, MA), cleaved caspase-8 (#9496, CST), GSDME (ab215191, Abcam, MA), ULK1 (#8054, CST), ULK2 (ab97695, Abcam), PARP (#9542, CST), p62 (ab56416, Abcam), LC3A/B (#12741S, CST) and β-actin (AF0003, Beyotime, Shanghai, China).

**Cell culture**

The leukemia cell lines THP-1, U937, Molt4, HEL92.1.7, K562, Raji, Jurkat, HL60 were grown in Roswell Park Memorial Institute 1640 medium (RPMI-1640) with 10% FBS and 1 × penicillin/streptomycin. All cells were grown at 37 °C in a 5% CO2 incubator. The cells have been regularly tested for potential mycoplasma contamination using the Myco-Blue Mycoplasma Detector (D101, Vazyme, Nanjin, China).

**RNA isolation, reverse transcription, and real time PCR**

Total RNA from indicated cells was isolated using NucleZOL (Macherey Nagel, Neumann) according to the manufacturer’s instructions. 1 μg total RNA was used for reverse transcription by HiScript® Ⅲ RT SuperMix with gDNA wiper (R323, Vazyme). Real time PCR was performed with ChamQ Universal SYBR qPCR Master Mix (Q711, Vazyme). β-actin was used as endogenous control transcripts for normalization of the target transcripts. Relative gene expression was analyzed using the 2-∆∆Ct method.

The primers used for human ULK1 (forward: AGCACGATTTGGAGGTCGC; reverse: GCCACGATGTTTTCATGTTTCA), and β-actin (forward: ACTGGAACGGTGAAGGTGAC; reverse: AGAGAAGTGGGGTGGCTTTT).

**Immunoblotting experiments**

The cells were treated with vehicle or compounds as described, then cells were collected by centrifugation with 500 g for 5 min, and washed twice in cold PBS and then lysed with RIPA Lysis Buffer (Beyotime). The lysates were quantified by BCA protein quantification kit (Yeasen). The proteins were separated by SDS-PAGE, immunoblotted, and visualized using Tanon 5200 Chemiluminescent Imaging System (Tanon Science & Technology).

**ATP cell viability assay**

As described previously [28]. The leukemia cells were plated in 48-well plates at about 3×10^5^ cells per well, and stimulated by indicated inhibitors. After that, the cells were collected and washed twice with PBS, and ATP level in the cells were determined by Enhanced ATP Assay Kit (S0027, Beyotime) according to the manufacturer’s instruction. 96-well black plates (Corning) for assaying ATP level were shaken on an orbital shaker for 2 min and incubated at room temperature for 10 min. Luminescence was then read using a Multi-Mode Microplate Reader (Varioskan Flash, Thermo Fisher Scientific Inc.). The dose inhibition curves and IC50 calculations for three ULK1 inhibitors were performed by Graphpad 7.0 software.

**Lentivirus Infection**

THP-1 and HL60 cells were seeded in 48-well plates at a density of 5 × 10^4^ cells per 500 µL, then transfected with lentiviral vector expressing short hairpin RNA (shRNA) against ULK1 or negative control (GenePharma, Suzhou, China) at a multiplicity of infection of 50 with polyprene at a concentration of 5 μg/ml. After 24 h, the culture medium was replaced according to the protocol provided by the manufacturer. At 72 h after transfection, ATP-based cell viability was analyzed by Enhanced ATP Assay kit. ULK1 expression in cells was detected by immunoblot to analyze knockdown efficiency. A lentivirus encoding short hairpin RNA (GenePharma) targeting ULK1 with the sequence 5′- GCACAGAGACCGTGGGCAA-3′ (LV3-ULK1) and negative control shRNA sequence 5′- TTCTCCGAACGTGTCACGT-3′ (LV3-NC) were constructed.

**Microscopy**

To examine the morphology of cell death, leukemia cells were seeded in 48-well plates at about 70%-80% confluence and then subjected to the indicated treatments. After that, static bright field cell images were captured using Leica AF6000 inverted microscope. The images shown are representative of at least three randomly selected fields.

**Caspase-3/8 activity experiments**

The THP-1 and HL60 cells were plated in 6-well plates at about 2×10^6^ cells per well, and stimulated by MRT68921 HCl. Once the stimulation ended, the caspase-3 and caspase-8 activity were assayed using Caspase-3 or Caspase-8 Activity Assay Kit (Beyotime) according to the manufacturer’s instructions.

**Autophagic flux analysis**

THP-1 and HL60 cells in the confocal dishes were transfected with mRFP-GFP-LC3 adenovirus (Hanbio, China) at a multiplicity of infection of 100. 24 h after transfection, cells were treated for 18 h with or without 5 µM MRT68921 HCl. Then, cells were scanned with a confocal microscope (SP8, Leica, Germany).

**NADPH/NADP^+^ ratio and ROS level measurement**

As described previously, THP-1 and HL60 cells were seeded in 12-well plates, and stimulated with MRT68921 HCl. After that, NADPH/NADP^+^ ratio and ROS level were determined by NADP^+^/NADPH Assay Kit with WST-8 and Reactive Oxygen Species Assay Kit (Beyotime), respectively. More specifically, cellular ROS level was measured using 2′,7′-dichlorodihydrofluorescein diacetate (DCFDA), and fluorescence-activated cell sorting (FACS) analysis, then median fluorescence intensity (MFI) quantification of ROS by using FlowJo software.

***In vivo* studies**

All mouse experiments were approved by the Institutional Animal Care and Use Committee of the Second Affiliated Hospital of Guangzhou Medical University. As described previously [28], Immunodeficient, non-obese, diabetic-severe, combined immunodeficient (NOD-SCID) IL2rg null (B-NDG) mice were obtaining from Beijing Biocytogen Co., Ltd. Female B-NDG mouse, specific pathogen free, with age of 6 weeks and body weight at about 20 g, were used for the experiments. Animals were on a balanced diet for rodents and given free access to water and food. THP-1 cells stably expressing the firefly luciferase gene were injected into the tail vein of 6-week-old female B-NDG mice (7 × 10^5^ cells per mouse). Seven days after injection, the mice were randomly grouped and treated intraperitoneally with 200 μl of vehicle (0.9% NaCl) or 200 μl MRT68921 HCl (30 mg/kg) once every two days for 3 weeks. Mice were weighed and observed daily. The tumor burden was measured on the desired days by bioluminescence imaging using Night Owl Ⅱ LB 983 system (Berthold, Germany). Survival analysis was measured from the time of cancer inoculation to the moribund state (six mice per group), and survival curves were plotted in Prism 7 (Graph Pad). Statistical significance was calculated using the log-rank test. Liver, spleen, and femur were harvested from three mice per group for paraffin embedding and hematoxylin/eosin (H&E) staining to analyze the leukemia progression. On day 21 of treatment, three mice were randomly picked up, and the liver, spleen, and bone marrow cells were collected and analyzed for the invasion of leukemia cells by FACS using anti-human-CD45 (#304012, Biolegend).

**Primary cell assays**

All primary leukemia cells specimens were derived from leftover diagnostic samples from patients, and this study was approved by Institutional Research Ethics Committee of the Second Affiliated Hospital of Guangzhou Medical University (approval number 2019-ks-29). Primary mononuclear cells (MNCs) derived from whole peripheral blood from leukemia patients were isolated using Lymphoprep™ and SepMate™-50 tubes (STEMCELL™ Technologies, Canada) and resuspended in RPMI-1640 containing 10% FBS. The red blood cells were then removed by ammonium chloride/potassium (ACK) lysis. All of these cells were cultured at 37℃ in a humidified incubator with 5% CO_2_. For cytotoxicity analysis, primary leukemia cells were plated in duplicate or triplicate in a 48-well plate at a density of 3×10^5^ cells per 500 μl per well. Each leukemia cells were treated with 5 μM MRT68921 HCl or vehicle (0.9% NaCl) and incubated for 24 h at 37℃, after that, the cells were collected and stained with Annexin V/PI (BestBio) to analyze cell death by flow cytometry.

**Statistical analysis**

All data were presented as mean ± SEM. Statistical software GraphPad Prism 7.0 was used for data analysis. All data were subjected to the two-side Student's *t*-test. Differences were considered statistically significant at *P* < 0.05.
