# Supplementary figures and images for: Inhibition of ULK1 promotes the death of leukemia cell in an autophagy irrelevant manner and exerts the antileukemia effect
Source: Clin Transl Med. 2021 Jan 12;11(1):e282. doi: 10.1002/ctm2.282 (PMC7803353; doi:10.1002/ctm2.282)

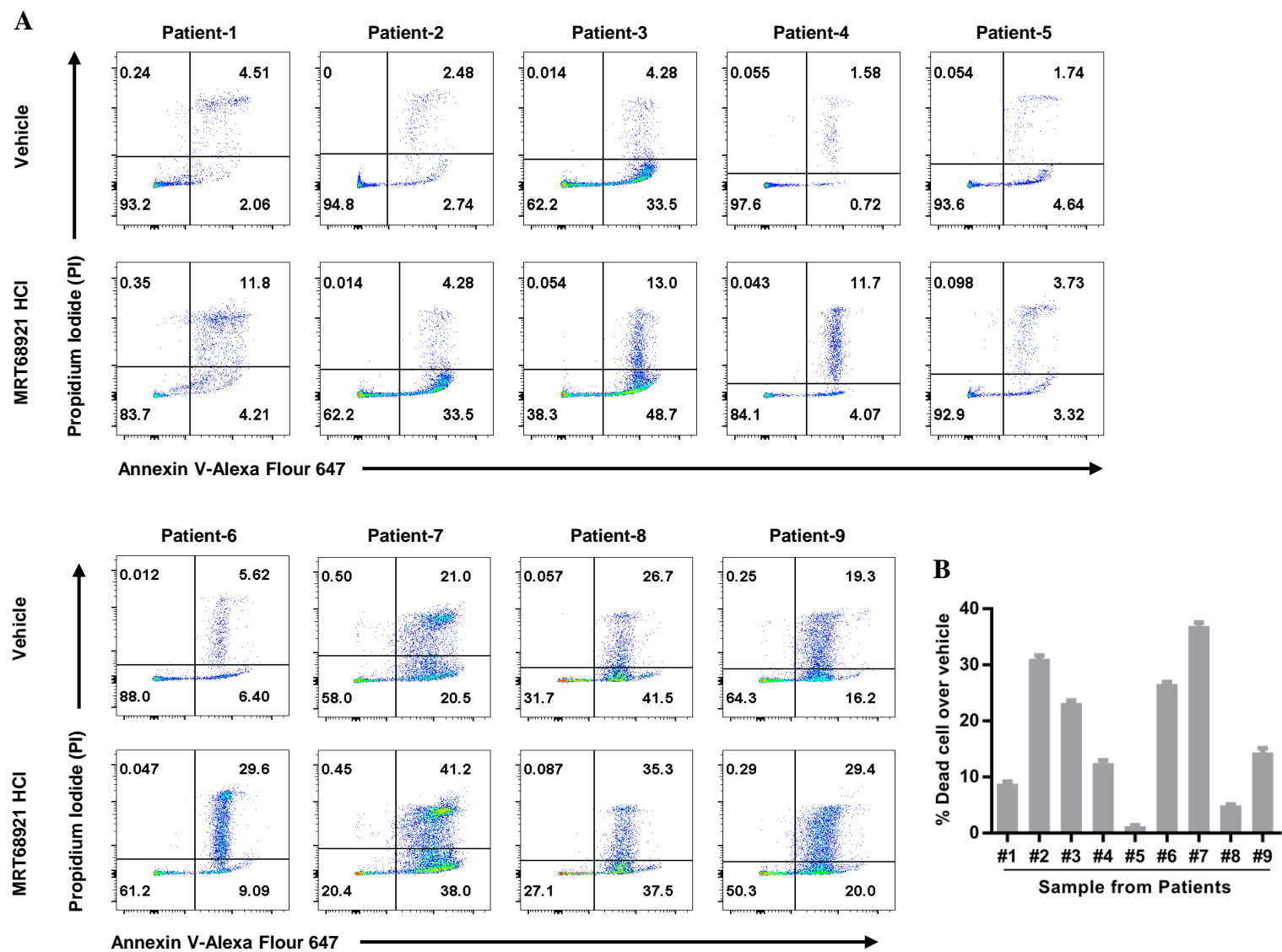

Supplement: Supplementary file 2 — Supporting Information [file CTM2-11-e282-s002.pdf]
